# Efficacy of Fluorecare SARS-CoV-2 Spike Protein Test Kit for SARS-CoV-2 detection in nasopharyngeal samples of 121 individuals working in a manufacturing company

**DOI:** 10.1371/journal.pone.0262174

**Published:** 2022-01-13

**Authors:** Valentina Tonelotto, Annamaria Davini, Laura Cardarelli, Milena Calderone, Paola Marin

**Affiliations:** 1 C.M.S.R. Veneto Medica S.r.l., Altavilla Vicentina, Vicenza, Italy; 2 Lifebrain S.r.l–Gruppo Cerba HealthCare c/o RDI—Rete Diagnostica Italiana S.r.l, Limena, Padova, Italy; FDA, UNITED STATES

## Abstract

**Objectives:**

The aim of this study was to evaluate the clinical performance of the Fluorecare SARS-CoV-2 Spike Protein Test Kit, a rapid immunochromatographic assay for SARS-CoV-2 detection. Moreover, we sought to point out the strategy adopted by a local company to lift the lockdown without leading to an increase in the number of COVID-19 cases, by performing a precise and timely health surveillance.

**Methods:**

The rapid Fluorecare SARS-CoV-2 Spike Protein Test was performed immediately after sampling following the manufacturer’s instructions. RT-PCRs were performed within 24 hours of specimen collection. A total amount of 253 nasopharyngeal samples from 121 individuals were collected between March 16 and April 2, 2021 and tested.

**Results:**

Of 253 nasopharyngeal samples, 11 (9.1%) were positive and 242 (90.9%) were negative for SARS-CoV-2 RNA by RT-PCR assays. The rapid SARS-CoV-2 antigen detection test’s mean sensitivity and specificity were 84,6% (95% CI, 54.6–98.1%) and 100% (95% CI, 98.6–100%), respectively. Two false negative test results were obtained from samples with high RT-PCR cycle threshold (Ct).

**Conclusion:**

Our study suggested that Fluorecare SARS-CoV-2 Spike Protein Test can be introduced into daily diagnostic practice, as its mean sensitivity and specificity follow the standards recommended by WHO and IFCC Task Force. In addition, we underlined how the strategy adopted by a local company to risk assessment and health surveillance was appropriate for infection containment. This real-life scenario gave us the possibility to experience potential approaches aimed to preserve public health and work activities.

## Introduction

The Coronavirus disease 2019 (COVID-19) pandemic firstly emerged in the city of Wuhan, China in December 2019 and rapidly spread across the world, causing dramatic clinical and socio-economic consequences [1–3]. The disease is caused by the severe acute respiratory syndrome coronavirus 2 (SARS-CoV-2) [4]. Since the discovery of SARS-CoV-2, huge efforts to develop suitable and high-throughput screening approaches have been made by academic laboratories and private companies. Among diagnostic routine procedures, reverse transcription quantitative real time-PCR (RT-PCR)-based analysis on nasopharyngeal (NP) swabs has been considered the gold standard diagnostic test [5, 6]. However, this approach has several limitations. Indeed, RT-PCR requires specialized instruments and skilled personnel. Moreover, it is a costly and time-consuming method that may delay patient management and the surveillance of virus transmission. Finally, due to economic challenges, RT-PCR may not be suitable for low and middle-income countries [7–9]. Therefore, a rapid and accurate diagnostic test for SARS-CoV-2 detection is necessary to promptly contain the spread of the disease.

Recent guidelines published by the World Health Organization (WHO) and the Task Force on COVID-19 of the International Federation of Clinical Chemistry and Laboratory Medicine (IFCC) [10, 11] have suggested the potential use of antigen rapid tests in biological materials, in particular NP samples and saliva, for mass testing. Antigen-based assays use monoclonal anti-SARS-CoV-2 antibodies, which recognize SARS-CoV-2 antigens, mainly N and S (Nucleocapsid protein and Spike surface glycoprotein, respectively) [12]. This technology has several advantages when compared to RT-PCR, such as the availability as a point of care (POC) diagnostic test, the lack of need for trained personnel and sophisticate instrumentation, lower costs, and a quick diagnostic response (5–30 min) [13]. Several commercial rapid immunoassays are now available [14], but there are few data on their clinical performance. Thus, analytical and clinical validation of these tests is fundamental before their introduction into daily diagnostic practice. Both the WHO and IFCC Task Force recommend the use of antigen assays with high specificity (i.e. ≥0.97) and acceptable sensitivity (i.e. ≥0.80) [10, 11]. Indeed, it is pivotal that such tests give reliable and reproducible results.

In this work, we report the results of our Fluorecare SARS-CoV-2 Spike Protein Test Kit-based screening, using NP specimens derived from 121 individuals all working in the same manufacturing company. Furthermore, we document the strategy adopted by a local company to lift the lockdown without leading to an increase in the number of COVID-19 cases. The cost-effective and timely health surveillance, distributed in a time period of less than 3 weeks, resulted in the identification of 11 (9.1%) positive samples among all coworkers. At the end of the surveillance period no positive samples were detected, suggesting that the strategy adopted by the employer, together with the work-occupational health physicians (OHP) and all those figures in charge of monitoring such measures, were appropriate for infection containment.

## Materials and methods

### Specimen collection

This investigation was carried out retrospectively, as part of clinical laboratory operations, using pre-existing specimens collected at Centro Medico Strumentale Riabilitativo (C.M.S.R.) Veneto Medica S.r.l. (Altavilla Vicentina, Italy). C.M.S.R. Veneto Medica S.r.l. is a medical center accredited with the National Health Service, providing different type of diagnostic services (e.g. cardiology, nuclear medicine, clinical pathology laboratory, diagnostic radiology). Samples collection was performed between March 16 and April 2, 2021, for the screening of SARS-CoV-2 infection in 121 individuals working in a local manufacturing company. A total amount of 253 samples were tested. Trained personnel collected two NP specimens, one of which (provided by the manufacturer) was used for the antigen testing, performed immediately upon specimens collection, while the other was placed in 3 mL of preservation medium (Jiansu Kangjjan Medical Apparatus, China) and used for the RT-PCR assays. The same specimen collection was adopted in [15].

### Fluorecare SARS-CoV-2 Spike Protein Test Kit

Among the commercially available kits we decided to adopt the Fluorecare kit according to manufacturer’s preliminary data. The Fluorecare® SARS-CoV-2 Spike Protein Test Kit (Microprofit Biotech, Shenzhen, China) exploits an immunochromatographic method to qualitatively detect the SARS-CoV-2 Spike Protein in nasal and NP swabs samples.

Briefly, the swabs were blended with a solution containing anti-SARS-CoV-2 Spike Protein antibodies which were fluorescently labeled. 60 μL of this solution were then deposited into the sample hole of the test card. If the Spike protein was present in the sample, it bound to the antibodies, forming a fluorescently-labeled complex. The complex diffused along the nitrocellulose membrane. Within the detection line area, the complex bound to the antibodies enclosed within this area, showing a red band under a fluorescent lamp. Fluorescently labeled SARS-CoV-2 antibodies also diffused to the quality control line (C) region and were captured by sheep anti-mouse IgG, to form red bands. These additional bands attested that the kit was working properly. A dedicated analyzer (Fluorecare MF-1000; Microprofit Biotech, Shenzen, China) was used for quantitative fluorescence reading. The final result was read within 15–20 minutes. The SARS-CoV-2 Spike Protein concentration was calculated by the preset calibration curve and the results were displayed with the unit of signal to cut-off (S/CO). The test was considered positive when the S/CO value was ≥1.2. Based on manufacturer’s indications, positive and negative percent agreement was 92.2% and 100%, respectively.

### Molecular testing

SARS-CoV-2 nucleic acid amplification was carried out using three different RT-PCR kits. The reason for using three different molecular kits was the limited supplied of reagents during the period in which this study was performed. In order to provide manageable results in a short time, and thus to fulfill the urgent need of containing the infection, RT-PCR tests were performed with kits for which reagents were immediately available. Each kit was validated by manufacturer and certified for *in vitro* Diagnostic use (CE-IVD) and we used it to address the need to identify SARS-CoV-2 positive samples.

The PerkinElmer® SARS-CoV-2 Real-time RT-PCR assay (PerkinElmer Inc. Waltham, USA) targets two SARS-CoV-2 specific genomic sequences, *N* and *ORF1ab*. TaqMan probes for the two RT-PCR products were labeled with FAM and HEX/VIC fluorescent dyes, respectively, in order to generate target-specific signals. Internal control RNA (IC, bacteriophage MS2) was also included in the test kit, to monitor the processes from nucleic acid extraction to fluorescence detection. A dUTP/UNG carryover prevention system was also used to avoid contamination of PCR products and subsequent false positive results. Results were considered positive when the cycle threshold (Ct) values of *N* and *ORF1ab* were lower than 33 and 37, respectively.

The REALQUALITY RQ-SARS-CoV-2 kit (AB Analitica, Padova, Italy) allowed to amplify the *RdRP* and *S* genes of SARS-CoV-2, respectively detected by FAM and JOE fluorescent dyes. Internal control RNA and dUTP/UNG system were also included in the kit. Results were considered positive when the Ct values of *RdRP* and *S* genes were lower than 38 and 35, respectively.

The MutaPLEX® Coronavirus Real-Time-RT-PCR (Immundiagnostik AG, Germany) kit contained specific primers and dual-labelled probes for the amplification of RNA of SARS-CoV-2 (*RdRP* gene and *S* gene, FAM channel; *N* gene, Cy5 channel) and the RNA of the Sarbecovirus subgenus (SARS-CoV-1 and SARS-CoV-2, *E* gene, Cy5 channel) extracted from biological specimens. The simultaneous detection of three SARS-CoV-2 targets including four gene sequences (*RdRP*, *S N* and *E*) increased the diagnostic reliability, even in cases of target sequence mutations. Furthermore, the kit contained a control RNA which was added during RNA extraction and detected in the same reaction by a HEX labelled probe and an Internal System Control (ISC). The ISC consisted of primers and probes for the detection of one housekeeping gene (*ACTB*, multi species) in the eluate from a biological specimen. The ISC helped preventing false negative results due to insufficient sample retrieval or transport. The amplification of the *ACTB* target sequence was measured in the ROX channel. Results were considered positive when the Ct values of *RdRP*/*S* were lower than 37 and the Ct values of *N* and *E* target sequences were lower than 33.

In this study, both the performance and reproducibility of the rapid SARS-CoV-2 antigen detection test were evaluated and compared with the RT-PCR test, which is considered the gold standard approach among COVID-19 screening methods.

### Statistical analysis

The diagnostic performance of Fluorecare SARS-CoV-2 Spike Protein Test Kit vs RT-PCR kits was evaluated by calculating the diagnostic sensitivity and specificity with MedCalc (MedCalc Software Ltd, Ostend, Belgium). This investigation was performed as part of clinical laboratory operations, using pre-existing specimens collected for routine SARS-CoV-2 diagnostics at the C.M.S.R. Veneto Medica S.r.l., thus patient informed consent and Ethical Committee approval were unnecessary. To preserve patient confidentially, samples were immediately de-identified and coded with a pre-assigned unique patient identifier. Samples were collected by trained nurses and then processed by the personnel in the clinical laboratories of C.M.S.R. Veneto Medica S.r.l. and Lifebrain S.r.l–Gruppo Cerba HealthCare c/o RDI—Rete Diagnostica Italiana S.r.l. All the results were anonymized. The study was conducted in accordance with the Declaration of Helsinki, under the terms of relevant local legislation.

## Results

### Characteristics of the screening

During the COVID-19 pandemic, each employment sector, together with OHP and other designated figures, adopted approaches for risk assessment and health surveillance connected to SARS-CoV-2 infection, in agreement with the guidelines issued by WHO, the European Centre for Disease Prevention and Control (ECDC) and the European Agency for Safety and Health at Work (EU-OSHA) [10, 16, 17]. The characteristics of the screening adopted to detect SARS-CoV-2 infection in our study are summarized in Table 1.

**Table 1:** Characteristics of the screening adopted by a local manufacturing company to detect SARS-CoV-2 infection between March 16 and April 2, 2021.

Total amount of people tested	121
Total amount of samples collected	253
Type of test	Molecular and rapid antigen test. NP specimens collected at the same time
Time period between each screening	Between 5 and 7 days
**First screening**	16–25 March 2021
Total amount of people tested during the first screening	121
Positive cases detected during the first screening	Rapid antigen test: 7
	Molecular test: 9
**Second screening**	25–26 March 2021
Total amount of people tested during the second screening	22
Positive cases detected during the second screening	Rapid antigen test: 2
	Molecular test: 2
**Third screening**	31 March-2 April 2021
Total amount of people tested during the second screening	110
Positive cases detected during the third screening	Rapid antigen test: 0
	Molecular test: 0

A total amount of 121 workers were tested for SARS-CoV-2 infection, between March 16 and April 2, 2021. Two NP specimens were collected for each patient, one for antigen testing, the other for the RT-PCR assays. A first screening of all the coworkers was performed between March 16 and March 25. 7 positive cases were detected with the antigen assay. The molecular test confirmed these 7 cases but identified 2 additional positive samples. Therefore, the first screening led to the identification of 9 positive cases, based on the RT-PCR results. These positive individuals were immediately placed in quarantine and isolation, and they were not re-tested in the two following screenings. 22 individuals, tested in the first screening and working in the same departments of the already identified positive cases, were then re-tested between March 25 and 26, with a time period between the first and second specimen collection of 5–7 days. This second screening allowed to identify 2 positive individuals, detected with both the antigen test and the molecular test. The 2 individuals were immediately placed in quarantine and they were not re-tested. A third screening was performed between March 31 and April 2, on the same cohort of individuals tested in the first and second screening (except for those resulted positive and still in isolation). No positive cases were identified in the third screening, neither with the antigen assay nor with the molecular test.

### Fluorecare SARS-CoV-2 Spike Protein Test Kit and RT-PCR kits

A single cohort of 121 patients was subjected to COVID-19 screening procedures in our laboratory. Among tested samples, 9 resulted positive with the Fluorecare SARS-CoV-2 Spike Protein Test Kit. The analysis performed with the RT-PCR kits confirmed these 9 positive cases, but identified 2 additional positive samples that were not detected with the antigen test. Therefore, the true positive cases among the cohort of 121 individuals were 11 (9,1%). The values of RT-PCR Ct and of Fluorecare SARS-CoV-2 Spike Protein Test Kit S/CO in positive and negative samples are summarized in Table 2, while the overall diagnostic performance of Fluorecare SARS-CoV-2 Spike Protein Test Kit is reported in Table 3.

**Table 2:** RT-PCR Ct values and Fluorecare SARS-CoV-2 Spike Protein Test Kit S/CO values in positive (A) and negative (B) samples.

RT-PCR assay	Ct	Result (based on RT-PCR assay)	Fluorecare SARS-CoV-2 Spike Protein Test Kit S/CO	Result (based on antigen assay)
**A**				
**PerkinElmer® SARS-CoV-2 Real-time RT-PCR assay (PerkinElmer**)	*N* gene = 18	Positive	9.54	Positive
	*ORF1ab* gene = 18			
	*N* gene = 19	Positive	>10	Positive
	*ORF1ab* gene = 19			
	*N* gene = 18	Positive	9.44	Positive
	*ORF1ab* gene = 21			
	*N* gene = 20	Positive	>10	Positive
	*ORF1ab* gene = 21			
**REALQUALITY RQ-SARS-CoV-2 kit (AB Analitica)**	*RdRP* gene = 27	Positive	7.42	Positive
	*S* gene = 27			
	*RdRP* gene = 19	Positive	>10	Positive
	*S* gene = 19			
	*RdRP* gene = 31	Positive	<1.2	Negative
	*S* gene = 31			
**MutaPLEX® Coronavirus Real-Time-RT-PCR (Immundiagnostik AG)**	*E* gene = 33	Weakly Positive	<1.2	Negative
	*RdRP/S* gene = 35			
	*E* gene = 24	Positive	1.2	Positive
	*RdRP/S* gene = 32			
	*E* gene = 18	Positive	9.52	Positive
	*RdRP/S* gene = 21			
	*E* gene = 18	Positive	>10	Positive
	*RdRP/S* gene = 21			
**TOTAL AMOUNT OF SAMPLES TESTED = 253**				
**TOTAL AMOUNT OF TRUE POSITIVE SAMPLES = 11**				
**B**				
**PerkinElmer® SARS-CoV-2 Real-time RT-PCR assay (PerkinElmer)**	*N* gene > 33	Negative	<1.2	Negative
	*ORF1ab* gene > 37			
**REALQUALITY RQ-SARS-CoV-2 kit (AB Analitica)**	*RdRP* gene > 38			
	*S* gene > 35			
**MutaPLEX® Coronavirus Real-Time-RT-PCR (Immundiagnostik AG)**	*E* gene > 33			
	*RdRP/S* gene > 37			
**TOTAL AMOUNT OF SAMPLES TESTED = 253**				
**TOTAL AMOUNT OF TRUE NEGATIVE SAMPLES = 242**				

**Table 3:** Clinical performance of Fluorecare SARS-CoV-2 Spike Protein Test Kit according to RT-PCR assays.

**Fluorecare SARS-CoV-2 Spike Protein Test Kit vs**	
**PerkinElmer® SARS-CoV-2 Real-time RT-PCR assay (PerkinElmer)**	
Sensitivity	100% (95% CI, 39.8–100%)
Specificity	100% (95% CI, 98.6–100%)
**Fluorecare SARS-CoV-2 Spike Protein Test Kit vs**	
**REALQUALITY RQ-SARS-CoV-2 kit (AB Analitica) assay**	
Sensitivity	75% (95% CI, 19.4–99.4%)
Specificity	100% (95% CI, 98.6–100%)
**Fluorecare SARS-CoV-2 Spike Protein Test Kit vs**	
**MutaPLEX® Coronavirus Real-Time-RT-PCR assay (Immundiagnostik AG)**	
Sensitivity	80% (95% CI, 28.4–99.5%)
Specificity	100% (95% CI, 98.6–100%)
**Fluorecare SARS-CoV-2 Spike Protein Test Kit vs**	
**RT-PCR assays**	
Sensitivity	84.6% (95% CI, 54.6–98.1%)
Specificity	100% (95% CI, 98.6–100%)

Four positive samples with a S/CO value >9 were confirmed as positive with the PerkinElmer SARS-CoV-2 Real-time RT-PCR assay, with Ct values of *N* and *ORF1ab* <21. Therefore, the sensitivity and specificity values of Fluorecare SARS-CoV-2 Spike Protein Test Kit, according to the Ct values obtained with the PerkinElmer RT-PCR kit, were both 100% (sensitivity: 95% CI, 39.8–100%; specificity: 95% CI, 98.6–100%).

Two positive samples with a S/CO value >7 also resulted positive when analysed with REALQUALITY RQ-SARS-CoV-2 kit, with Ct values <27 for *RdRP* and *S* genes. One sample with a S/CO value <1,2 and thus classified as negative with the antigen test, was instead detected as positive with the AB Analitica RT-PCR kit, with Ct values of 31 for *RdRP* and *S* genes. Accordingly, when calculated on the basis of the results obtained with the AB Analitica RT-PCR kit, the sensitivity and specificity values of Fluorecare SARS-CoV-2 Spike Protein Test Kit were 75% (95% CI, 1.4–99.4%) and 100% (95% CI, 98.6–100%), respectively.

The MutaPLEX Coronavirus Real-Time-RT-PCR kit confirmed 3 positive samples first detected with the antigen test. In particular, 2 samples with a S/CO value >9.5 were confirmed as positive with a Ct value of 21 for *RdRP* and *S* genes and a Ct value of 18 for *E* gene. The third sample exhibited a S/CO value = 1.2 and was confirmed as positive with a Ct value of 32 for *RdRP* and *S* and a Ct value of 24 for *E*. On the other hand, one sample detected as negative (S/CO value <1.2) with the Fluorecare SARS-CoV-2 Spike Protein Test Kit, was instead classified as weakly positive with the MutaPLEX Coronavirus Real-Time-RT-PCR kit, with a Ct value of 35 for *RdRP* and *S* and a Ct value of 33 for *E*. Therefore, the sensitivity and specificity values of Fluorecare SARS-CoV-2 Spike Protein Test Kit, when determined considering the results obtained with the MutaPLEX Coronavirus Real-Time-RT-PCR kit, were 80% (95% CI, 28.4–99.5%) and 100% (95% CI, 98.6–100%), respectively.

When compared to all the RT-PCR assays used in this study, the overall sensitivity and specificity of Fluorecare SARS-CoV-2 Spike Protein Test Kit resulted 84.6% (95% CI, 54.6–98.1%) and 100% (95% CI, 98.6–100%), respectively.

## Discussion

In the rapidly evolving situation of COVID-19 pandemic, the availability of high throughput and appropriate methods for SARS-CoV-2 infection screening and diagnosis should be considered as a priority. In this scenario, the adoption of rapid antigen immunoassays as alternative to laborious RT-PCR-based procedures has been proposed as a valid and prompt solution. In our study, a cohort of 121 individuals working in the same manufacturing company was tested for SARS-CoV-2 infection. We exploited the Fluorecare SARS-CoV-2 Spike Protein Test Kit and evaluated its clinical performance by comparison to RT-PCR assays. Overall, the mean sensitivity and specificity of the antigen assay resulted 84.6% and 100%, respectively, indicating that this assay follows the standards recommended by WHO and IFCC Task Force (sensitivity ≥80%, specificity ≥97%). However, the few positive cases identified in this study, together with the fact that different RT-PCR assays were used, may influence the evaluation of the real diagnostic performance of Fluorecare SARS-CoV-2 Spike Protein Test Kit. Indeed, Salvagno et al. [18] performed a clinical evaluation of this specific antigen assay on 354 individuals, 233 of whom were positive at RT-PCR, and demonstrated that the sensitivity highly varies depending on Ct values. The authors revealed that the overall sensitivity of the assay was relatively modest (27.5%), but it became 90.5% when considering Ct <25. These results led the authors to conclude that in presence of samples with high viral load (i.e. Ct values <25) Fluorecare SARS-CoV-2 Spike Protein Test Kit could be a suitable test for screening of patients with higher infective potential [18].

The low number of positive cases in our study did not allow to stratify the antigen assay performance according to Ct values. Nevertheless, it is interesting to notice that one sample classified as negative, because the S/CO was <1.2, resulted instead weakly positive when analysed with MutaPLEX Coronavirus Real-Time-RT-PCR kit, with a Ct value of 35 for *RdRP/S* and of 33 for *E*. Since samples with a Ct value of *E* >33 were classified as negative, this result suggested that this specific sample may had a low viral load, that was not detected by the antigen assay but was barely detected by RT-qPCR. The same is true for another sample resulted negative at the antigen assay but classified as positive with the REALQUALITY RQ-SARS-CoV-2 kit, with Ct values of 31 for both *RdRP* and *S*. Therefore, Fluorecare SARS-CoV-2 Spike Protein Test Kit effectively seems a reliable screening test for patients with high infective potential, as demonstrated also by the correspondence between high S/CO values (>9) and low Ct values of *N* and *ORF1ab* (<21) obtained with PerkinElmer SARS-CoV-2 Real-time RT-PCR assay. However, borderline S/COs should not be underestimated, as we identified a positive sample with a S/CO = 1.2 which was then confirmed as positive with MutaPLEX Coronavirus Real-Time-RT-PCR kit. Concerning the specificity, in our study this parameter resulted 100%, as declared by the manufacturer. Indeed, no false positive samples were detected.

An important key aspect is that the low number of detected positive cases was the result of the strategies adopted to contain the infection transmission. The first screening of the 121 coworkers allowed to identify 9 positive individuals (based on the RT-PCR results) that were immediately isolated and put in quarantine. The employer, together with the OHP and the safety manager, adopted all the recommended procedures to prevent the emergence of new epidemic clusters, such as temperature monitoring in the workplace, timely laboratory testing strategies, contact tracing and teleworking [1, 2]. This led to a second screening of 22 individuals, already tested, that worked in the same sectors of the identified positive people, resulting in the identification of 2 additional positive specimens. The measures to contain the spreading of the infection were further reinforced and, in a third screening of all still negative individuals, performed between 5 and 7 days after the second screening, no positive samples were detected.

Overall, this study underlines how the cooperation between employers and all relevant health figures (OHPs, nurses, clinicians, biologists) and the adoption of appropriate tests for diagnosis are fundamental to contain the dramatic consequences of a pandemic such as COVID-19. This real-life scenario gave us the possibility not only to evaluate the clinical performance of Fluorecare SARS-CoV-2 Spike Protein Test Kit, a new antigen assay, but also to experience the success of a prompt and clever strategy on preserving public health and work activities.
